# Tachyarrhythmia in patients with congenital heart disease: inevitable destiny?

**DOI:** 10.1007/s12471-015-0797-z

**Published:** 2016-01-04

**Authors:** C. P. Teuwen, Y. J. H. J. Taverne, C. Houck, M. Götte, B. J. J. M. Brundel, R. Evertz, M. Witsenburg, J. W. Roos-Hesselink, A. J. J. C. Bogers, N. M. S. de Groot, DANARA Study Investigators

**Affiliations:** 1 Department of Cardiology, Erasmus University Medical Center, ‘s Gravendijkwal 230, 3015 CE Rotterdam, The Netherlands; 2 Department of Cardio-Thoracic Surgery, Erasmus University Medical Center, Rotterdam, The Netherlands; 3 Department of Cardiology, Haga Hospital, The Hague, The Netherlands; 4 Department of Clinical Pharmacy and Pharmacology, University Medical Center Groningen, Groningen, The Netherlands; 5 Department of Physiology, Institute of Cardiovascular Research, VU University Medical Center, Amsterdam, The Netherlands; 6 Department of Cardiology, University Medical Center St. Radboud, Nijmegen, The Netherlands

**Keywords:** Congenital heart defects, Cardiac surgery, Atrial tachyarrhythmia, Ventricular tachycardia, Catheter ablation

## Abstract

The prevalence of patients with congenital heart disease (CHD) has increased over the last century. As a result, the number of CHD patients presenting with late, postoperative tachyarrhythmias has increased as well. The aim of this review is to discuss the present knowledge on the mechanisms underlying both atrial and ventricular tachyarrhythmia in patients with CHD and the advantages and disadvantages of the currently available invasive treatment modalities.

## Introduction

Congenital heart disease (CHD) is defined as a developmental malformation of the heart chambers, valves or great vessels. The incidence of newborns with CHD has increased over the last century from 0.6 per 1000 live births in 1930, to 9.1 per 1000 live births after 1995, thereby making CHD a major public health issue [1]. This development is caused by more accurate registration procedures and improved diagnostic tools (e.g. cardiac imaging techniques). However, there are still geographical differences in the prevalence of CHD birth rates, which can be explained by e.g. genetic or environmental factors [1]. The number of *adult* CHD patients has also increased in the past decades, as nowadays over 90 % of paediatric patients survive into adulthood due to improved clinical care and surgical techniques [2]. Although survival of CHD patients has been significantly prolonged, many of them frequently experience complications such as rhythm disorders by the time they reach adulthood [3]. These postoperative dysrhythmias may cause a wide range of symptoms, ranging from palpitations to even sudden cardiac death.

Many of these late postoperative tachyarrhythmias are, however, insufficiently controlled by antiarrhythmic drugs [4]. A lifetime usage of class III antiarrhythmic drugs such as amiodarone may result in less recurrences [5], but also increases the risk of adverse effects in the relatively young adult CHD patient, particularly in women with CHD, cyanotic patients and patients with a Fontan circulation [6]. Atrial pacing in order to prevent tachyarrhythmias is often not effective [7]. However, endovascular catheter ablation has arisen since the 1990s and both short- and long-term outcomes are promising [8].

Most studies reporting on late postoperative tachyarrhythmias in CHD patients described the incidence of the various types of tachyarrhythmia, the outcome of different treatment modalities, and in case of ablative therapy, the mechanism of the tachyarrhythmia and the location of successful target sites for catheter ablation in small groups of patients with a variety of CHD. The purpose of this review is to outline the present knowledge of the mechanisms underlying atrial and ventricular tachyarrhythmia in CHD patients and to discuss the advantages and limitations of the currently available invasive treatment modalities.

## Atrial macro reentrant tachycardia

**Fig. 1:**
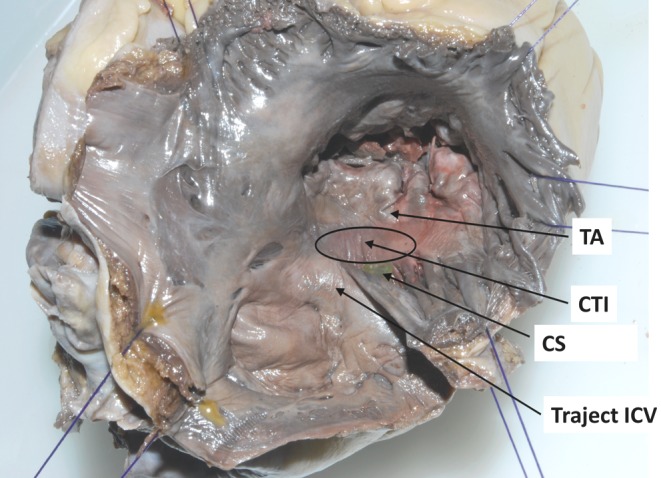
Anatomy of the cavotricuspid isthmus. Postmortem human heart with a superolateral view of the right atrium (turned inside out) with a bicaval incision. The cavotricuspid isthmus, which is regarded as the zone of slow conduction, is encircled. The isthmus is bordered anteriorly by the TA and posteriorly by the orifice of the ICV. *CS* coronary sinus, *CTI* cavotricuspid isthmus, *ICV* inferior vena cava, *TA* tricuspid annulus

**Fig. 2:**
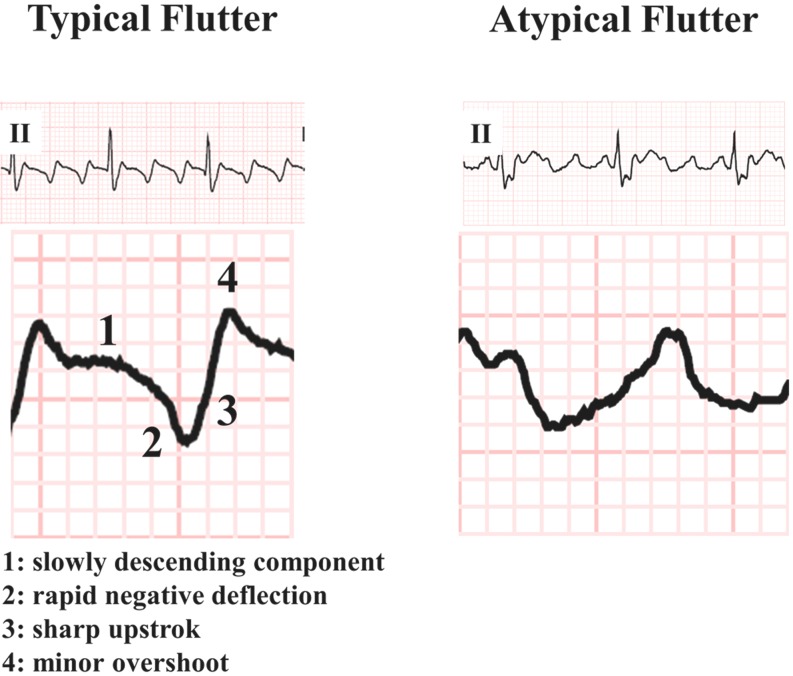
ECG characteristics of regular atrial tachycardias. *Left panel*: typical atrial flutter consisting of flutter waves with *1*) flat descending part, *2*) steep descending transition, *3*) sharp upstroke and *4*) a minor overshoot. *Right panel*: intra-atrial reentrant tachycardia; the four characteristics of the typical flutter waves are missing

Atrial macro-reentrant tachycardias are the most frequently reported atrial tachyarrhythmias in patients with both repaired and unrepaired CHD. They can be classified as either an intra-atrial reentrant tachycardia (IART) or typical clockwise and counterclockwise atrial flutter (AFL) which also occurs in patients without CHD [3, 9–12]. Most macro-reentry circuits in CHD patients are located within the right atrium [8]. The incidence of typical AFL has mainly been observed in patients with tetralogy of Fallot (ToF) or atrial septal defect (ASD) [10, 12–14]. AFL is caused by a macro-reentrant circuit located within the right atrium (Fig. 1) and it is bordered by the tricuspid annulus (anteriorly), the orifices of the superior and inferior caval vein (Eustachian ridge, posteriorly), the coronary sinus and the crista terminalis. The smallest pathway within the reentry circuit is the cavotricuspid isthmus, which is often a zone of slow conduction. Typical counterclockwise AFL waves on the surface electrocardiogram (left panel of Fig. 2) consist of a slowly descending component, rapid negative deflection, sharp upstroke and minor overshoot [15]. Catheter ablation is aimed at creating a linear lesion across the cavotricuspid isthmus which establishes a line of conduction block which in turn interrupts the reentrant wavelet.

All other atrial reentry tachycardias, not using the reentry circuit of typical AFL in either the right or left atrium, are defined as IART and have frequently been described in patients with a univentricular heart and transposition of the great arteries (TGA) [12, 16]. The cavotricuspid isthmus may still be part of the reentry circuit, but the reentry wavelet may circulate around other structures, such as areas of scar tissue, surgically inserted material or suture lines [17]. The reentrant wavelet in the atria of CHD patients can often follow different pathways due to the presence of multiple corridors between patchy areas of scar tissue, anatomical structures or surgically inserted material [18]. As demonstrated by the surface ECG in the right panel of Fig. 2, the four characteristics of typical flutter waves are usually not present.

**Fig. 3:**
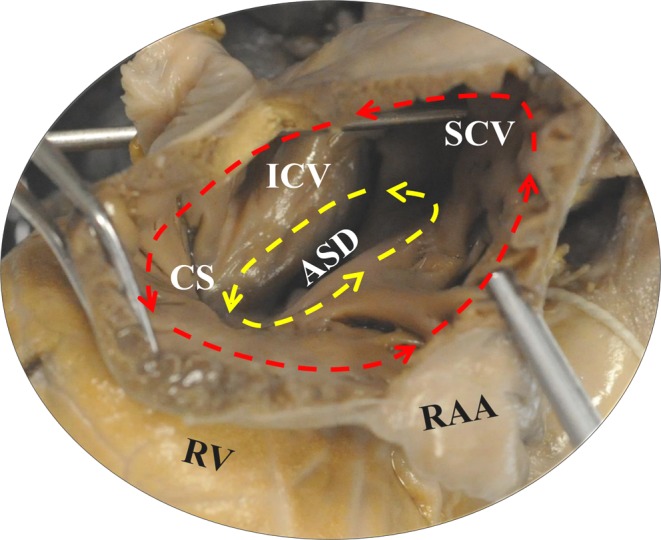
Anatomy of heart with CHD. Postmortem human heart of a 4-year-old child with a large atrial septal defect. The heart is shown from a lateral view through a right atriotomy incision into the right atrium. In adult patients with congenital heart defects, the intra atrial reentry tachycardia is frequently observed around the right atriotomy scar (*red marked area*), but also around the atrial septal defect (*yellow marked area*). *ASD* atrial septal defect, *CS* coronary sinus, *ICV* inferior caval vein, *RAA* right atrial appendage, *RV* right ventricle, *SCV* superior caval vein

Reentry pathways of IARTs described in literature are highly variable. The right atriotomy scar, creating crucial pathways of conduction between the right atriotomy site and the inferior caval vein, is often involved in IART [10–12, 18]. In patients with a univentricular heart or TGA, areas of slow conduction have been found along inserted prosthetic materials such as the Fontan conduit or intra-atrial baffles after the Senning or Mustard procedure [8, 10, 18]. Furthermore, regions around the septal patch in patients with ASD after surgical correction commonly function as crucial pathways of conduction (Fig. 3).

Reentry circuits have also been found in the left atrium, though less frequently. They have been observed in patients with ASD, TGA, univentricular heart and ToF, but descriptions of the exact pathways have not been given [12, 18].

Although an ECG might provide a clue about the pathway of the reentrant wavelet, invasive electrophysiological studies are essential to determine the underlying mechanism of the arrhythmia and to identify the crucial pathway of conduction [19]. Endovascular catheter ablation is then aimed at transecting this pathway in order to terminate the tachyarrhythmia.

Initial ablation procedures of postoperative atrial tachycardia in CHD patients were guided by fluoroscopy only [20]. Target sites for ablation were solely selected by using entrainment mapping techniques. However, selection of the appropriate target site for ablation was difficult as it required an imaginary three-dimensional (3D) reconstruction of the (multiple) reentrant circuits in a complex cardiac anatomy. The success rate often depended on the complexity of the underlying heart defect [8, 20].

**Fig. 4:**
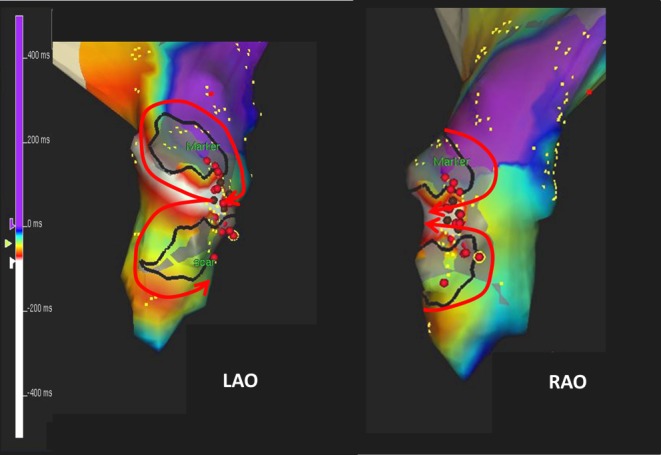
Electroanatomical mapping of IART. Three-dimensional electroanatomical mapping of the right atrium in a 15-year-old patient, 12 years after completion of the Fontan correction, who was referred for ablative therapy of an incessant atrial tachycardia. The colour-coded right atrial activation map shows a figure-of-eight reentry around 2 areas of scar tissue. The tachycardia was eliminated by constructing a linear lesion between 2 areas of scar tissue

The introduction of 3D electroanatomical mapping techniques enabled 3D visualisation of the patterns of activation (Fig. 4), thereby facilitating selection of appropriate target sites for ablation. The use of this technology resulted in improved outcomes of ablative therapy [18]. In addition to this, new techniques facilitated navigation to the target site and the usage of irrigated tip catheters improved lesion formations and further increased the success rate [21–23]. Although catheter ablation with a success rate of 90 % has been reported, ablation of IART is less successful than that of AFL. This may be due to e.g. insufficient lesion depth in the thickened atrial wall or conversion from one atrial tachycardia to another during ablation due to the presence of multiple pathways.

Despite successful procedural outcome of catheter ablation, atrial tachycardia recurs frequently. The reentry circuit and subsequently the crucial pathway of conduction may be located at the same site of the previous ablation [8], but they have often been found at other sites [24]. Recurrences of atrial tachycardia may also be caused by different mechanisms. For example, a focal atrial tachycardia may develop after successful ablation of IART [24]. The arrhythmogenic substrate of recurrences was often located at other atrial sites, indicating that the atrial tachycardia was not related to the previous tachycardia. These ‘recurrent’ tachycardias after ablative therapy may simply reflect a progressive cardiomyopathy caused by the persisting pressure/volume overload in CHD patients after cardiac surgery. This on-going remodelling process affects intra-atrial conduction, thereby creating a new arrhythmogenic substrate facilitating development of other tachyarrhythmias.

## Focal atrial tachycardia

**Fig. 5:**
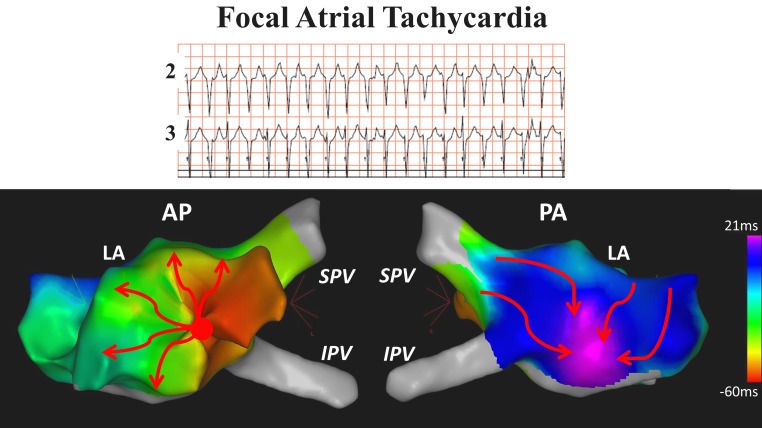
Electroanatomical mapping of focal atrial tachycardia. A 17-year -old patient with patent foramen ovale presented with paroxysmal episodes of regular atrial tachycardia. During an invasive electrophysiological study with 3D activation mapping, the atrial tachycardia (cycle length 348 ms) had a focal origin at the left atrial free wall. The map shows expansion from one circumscribed area in the anterior-posterior view (*AP*) to the remainder of the atrium in the posterior-anterior view (*PA*). After construction of a circular lesion around the earliest activated area, the tachycardia terminated

Focal atrial tachycardias are defined as arrhythmias originating from a small, circumscribed area from where it expands to the remainder of the atria and have been observed in various types of CHD (Fig. 5; [10, 11, 13]).

Expansion of the wavefront from its site of origin through multiple areas of conduction delay can bridge the diastolic interval thereby giving rise to flutter waves on the surface ECG. Hence, differentiation between a focal atrial tachycardia and an IART may be difficult using the surface ECG only and invasive electrophysiological studies are therefore crucial to correctly diagnose the underlying mechanism.

Several studies demonstrated that the origins of focal atrial tachycardia were located along the borders of areas of scar tissue. Although areas of scar tissue are found scattered throughout both the right and left atrium in patients with CHD, they mainly originate from the right atrium [10, 24].

Theoretically, focal atrial tachycardia can be caused by enhanced automaticity, triggered activity or micro-reentry [25]. De Groot et al. observed prolonged fractionated potentials at the origins of focal atrial tachycardia reflecting local dissociation in conduction suggestive of micro-reentry as the underlying mechanism [10].

The success rate of ablative therapy of focal atrial tachycardia in patients with a variable complexity of CHD was high (86–100 %) [10, 26]. However, comparable with atrial reentry tachycardia, ‘recurrences’ of atrial tachyarrhythmia after ablation of focal atrial tachycardia have been reported. Most atrial tachycardia developed within three years or even less and was mainly caused by other mechanisms (e.g. IART) [24].

## Atrial fibrillation

Atrial fibrillation (AF) is less frequently observed in CHD patients than regular atrial tachycardia [27, 28]. Whereas ablative therapy is nowadays an accepted treatment modality for regular atrial tachycardia in CHD patients, endovascular catheter ablation of AF in CHD patients is less well established. In addition to this, it is unknown whether the mechanism underlying AF in CHD patients is comparable with patients without CHD. The lifetime pressure and stretch may lead to sinus node dysfunction and increased ectopy (triggers) that initiate atrial tachyarrhythmias [29]. The overload may result in fibrosis and thereby conduction disorders which are likely to form a substrate for arrhythmias such as AF and regular atrial tachycardia. In a recent study including 199 patients with various CHD, it was indeed shown that AF and regular atrial tachycardia co-exist [27]. De Groot et al. found that a surface ECG resembling AF in two patients was the result of continuous electrical activity within a circumscriptive area at the right atrial posteroseptal and the anterolateral free wall [10]. Isolation of these areas by ablative therapy terminated AF. In line with these findings, Takahashi et al. also demonstrated that AF was the result of continuous fractionated electrical activity in the right atrial free wall and lower interatrial septum [30]. After ablation of these sites, the patient converted to sinus rhythm.

Endovascular pulmonary vein isolation (PVI) has been described in a limited number of patients. Four patients with an ASD and either paroxysmal (*N* = 2) or persistent (*N* = 2) AF were scheduled for percutaneous closure of the ASD [31]. Prior to closure, endovascular PVI was performed in all 4 patients; additional lesions were created in the 2 patients with persistent AF including a circular lesion around the superior caval vein and a linear lesion connecting the right and left pulmonary veins and mitral isthmus line. A recurrent AF episode occurred in only 1 patient after a follow-up period of 21 months in the early postoperative period after an orthopaedic surgical operation and was controlled with antiarrhythmic drug therapy (dronedarone). Philip et al. performed PVI in 36 patients with CHD (ASD, ventricular septal defect (VSD), ASD and VSD, ToF, double outlet left ventricle and TGA, coarctation of the aorta, Ebstein anomaly, and Bland-Garland White syndrome) with paroxysmal (*n* = 26) or persistent (*n* = 10) AF. After a mean follow-up period of 4 years, freedom of AF was achieved in 27 % [32].

In patients with CHD and AF, the Cox-Maze technique has been applied since the 1990s. A right-sided Maze procedure was performed in 77 CHD patients with preoperative AF (left atrial size < 41 mm) [33]. After a follow-up period of 2.7 years, 90 % (*n* = 56) of the patients were free from AF. However, other studies showed higher recurrence rates of AF in CHD patients who underwent only a right-sided Maze procedure compared with patients with a right- and left-sided Maze. Im et al. reported sinus rhythm without episodes of atrial tachyarrhythmias or pacemaker implantation in 69 % of the patients with right- and left-sided Maze procedure after 5 years of cardiac surgery compared with only 45 % of the patients with a right-sided Maze [34]. Moreover, recurrences of AF seem to be rare in other studies when antiarrhythmic surgery includes the right and left atrium, suggesting that the left atrium plays a (major) role in the pathophysiology of AF in patients with CHD as well [35]. Altogether, a concomitant Maze procedure should be considered in CHD patients known with AF who undergo corrective/palliative surgery at adult age.

## Atrioventricular reentry tachycardia

Although less common than other supraventricular tachycardia, atrioventricular reentry tachycardia (AVRT) due to accessory bundles in CHD patients has been described, especially in patients with Ebstein anomaly [36]. Moreover, approximately half of these patients have multiple accessory bundles which often have antegrade and retrograde conduction. Antegrade fast conduction during atrial tachyarrhythmias can lead to life-threatening arrhythmias of the ventricles. Catheter ablation is used to interrupt the accessory pathway in both children and adults with CHD. However, the possibility of multiple accessory bundles and defiant morphology of the heart with abnormal endocardial electrograms makes successful ablative therapy more challenging [36]. If catheter ablation is unsuccessful, surgical treatment of the accessory bundles might be an alternative [37].

## Ventricular tachycardia

Ventricular tachycardia (VT) also develops in patients with CHD, although with a lower prevalence than atrial tachyarrhythmias. Scars in the ventricular wall caused by surgical procedures or implantation of septal patches may form borders of complex reentry circuits thereby facilitating development of reentry tachycardias [38]. However, VT also occurs in CHD patients who have not undergone surgery [39]. Therefore, next to suture lines impairing ventricular conduction, other mechanisms may be involved as well. Structural alterations such as increment in fibrotic tissue or myocyte hypertrophy due to volume overload may result in conduction abnormalities, giving rise to VT [40, 41]. Cardiac magnetic resonance imaging can be useful to identify the substrate underlying the VT [42].

VT have mainly been described in patients with ToF, but also in patients with other CHD such as aortic valve disease, pulmonary valve stenosis, VSD and TGA [40, 43]. The consequences of VT are severe and may result in syncope and even sudden cardiac death. Effective management of this tachyarrhythmia is therefore essential. According to the European guidelines, an implantable cardioverter defibrillator (ICD) is indicated and recommended in patients with ventricular fibrillation or sustained VT with unsuccessful catheter ablation therapy [44]; earlier studies have shown that appropriate shocks occur in around 25–30 % of these CHD patients with an ICD [45]. Unfortunately, inappropriate shocks occur frequently as well (up to 40 %) [45]. On top of that, an ICD implantation appears to have a great impact on the quality of life in these patients [46]. Primary prevention of sudden cardiac death remains challenging and is mostly based on multiple additional determinants such as increased QRS duration and depressed ventricular function. There is no evidence that programmed ventricular stimulation predicts sudden cardiac death; however, it may be valuable in patients with ToF [47].

Invasive electrophysiological studies have been performed in order to locate the substrate of VT in CHD patients with e.g. ToF and VSD [40, 43]. These studies demonstrated that crucial pathways were indeed often bordered by unexcitable tissue around surgically corrected areas such as the infundibulotomy scar, right ventricular outflow tract and ventricular septal patch. Although left-sided VT has also been reported [48], VT in these patients has mainly been observed to originate from the right ventricle.

Gonska et al. reported acute procedural successful outcome of ablative therapy of 94 %, using fluoroscopy-guided catheter ablation [40]. It is likely that, comparable with atrial reentry tachycardia, the introduction of 3D electroanatomical mapping technique facilitated identification of reentrant pathways, leading to improved outcomes of ablative therapy [43]. Zeppenfeld et al. performed 3D electroanatomical mapping studies and subsequently ablative therapy in 11 CHD patients [43]. They achieved non-inducibility of all VTs (*N* = 15), including ablative therapy of haemodynamically unstable VTs that were guided by sinus rhythm mapping only. However, Morwood et al. reported an acute success rate of only 50 %, caused by either non-inducibility of the clinical VT or induction of haemodynamically unstable VT [49].

As for the long-term success, Gonska et al. observed recurrences in 20 % of the successfully ablated patients (*N* = 15) after a follow-up period of 16 ± 9 months [40]. Zeppenfeld et al. did not document any recurrences in the 11 patients but an ICD was implanted in 1 patient because of inducibility of sustained VT during a second electrophysiology study [43]. Comparable with CHD patients with atrial tachyarrhythmias, surgical ablation is possible in CHD patients with VT [50]. During the operation, VT can be induced after which mapping is subsequently possible in order to locate the substrate of VT and perform cryo-ablation. Previous studies showed considerable success rates of cryo-ablation with a 3-year VT recurrence-free survival of 80 % [50].

## DANARA (Dysrhythmia in pAtients with congeNital heARt diseAse)

In summary, the high incidence of tachyarrhythmia in ageing patients with CHD and the improved mapping techniques over the years went hand-in-hand with increased knowledge of the underlying mechanism and improved outcome of ablative therapy. In patients with haemodynamically unstable tachyarrhythmias or patients with symptoms and drug-refractory tachyarrhythmias, catheter ablation should be considered. The possibility of multiple arrhythmias and previous, failed invasive procedures should be taken into account in order to estimate the success rate of ablative therapy. Yet, after successful ablative therapy new tachycardias continue to develop. Insight into the development of these recurrent tachycardias is essential in order to develop preventive strategies. Another challenge is to elucidate the mechanism of AF in this study population, as the incidence of AF continues to rise in this ageing population. In order to gain further insight into the pathophysiology of dysrhythmias, we initiated an international multicentre study (DANARA project) focussing on development of dysrhythmias in patients with CHD by correlating the occurrence of arrhythmias over time in relation to clinical profiles. In addition, we perform intra-operative high resolution mapping studies in order to examine the arrhythmogenic substrate. With this project, we hope to improve our comprehension of these complex, but ever-challenging arrhythmias.

### Disclosures

Dr. B.J.J.M. Brundel is supported by the LSH-Impulse grant (40-43100-98-008) and the Dutch Heart Foundation (2013T144, 2013T096 and 2011T046) and Dr. N.M.S. de Groot is supported by grants from the Erasmus Medical Center fellowship, Dutch Heart Foundation (2011T046), CoolSingel foundation (project no. 212), LSH-Impulse grant (40-43100-98-008), Bayer and Boehringer Ingelheim.

### Conflict of interests

None declared.
