# Erratum to: Tachyarrhythmia in patients with congenital heart disease: inevitable destiny

**DOI:** 10.1007/s12471-016-0823-9

**Published:** 2016-03-08

**Authors:** C. P. Teuwen, Y. J. H. J. Taverne, C. Houck, M. Götte, B. J. J. M. Brundel, R. Evertz, M. Witsenburg, J. W. Roos-Hesselink, A. J. J. C. Bogers, N. M. S. de Groot, DaNaRa Study Investigators

**Affiliations:** 1 Department of Cardiology, Erasmus University Medical Center, Rotterdam, The Netherlands; 2 Department of Cardio-Thoracic Surgery, Erasmus University Medical Center, Rotterdam, The Netherlands; 3 Department of Cardiology, Haga Hospital, The Hague, The Netherlands; 4 Department of Clinical Pharmacy and Pharmacology, University Medical Center Groningen, Groningen, The Netherlands; 5 Department of Physiology, Institute of Cardiovascular Research, VU University Medical Center, Amsterdam, The Netherlands; 6 Department of Cardiology, University Medical Center St. Radboud, Nijmegen, The Netherlands

**Erratum to**: Neth Heart J (2016)

DOI 10.1007/s12471-015-0797-z

In the version of the article originally published online, the members of the DANARA study group were appended to the list of authors. The author line should have read:

Christophe P. Teuwen, MD^1^; Yannick J.H.J. Taverne, MD^2^; Charlotte Houck, Msc^1^; Marco Götte, MD, PhD^3^; Bianca J.J.M. Brundel, PhD^4,5^; Reinder Evertz, MD^6^; Maarten Witsenburg, MD, PhD^1^; Jolien W. Roos-Hesselink, MD, PhD^1^; Ad J.J.C. Bogers, MD, PhD^2^; Natasja M.S. de Groot, MD, PhD^1^; DANARA Study Investigators

Collaborators: Sander G. Molhoek, MD, PhD^7^; Tanwier T.T.K. Ramdjan, MSc^1^; Wim A. Helbing, MD, PhD^8^; Janneke A.E. Kammeraad, MD, PhD^8^; H.G. Reinhart Dorman, MD^9^; Jurren M. van Opstal, MD, PhD^10^; Thelma C. Konings, MD^11^; Joris W.J. Vriend, MD, PhD^3^; Pepijn van der Voort, MD^12^;

^1^Dept of Cardiology, Erasmus University Medical Center, Rotterdam, The Netherlands; ^2^Dept of Cardio-Thoracic Surgery, Erasmus University Medical Center, Rotterdam, The Netherlands; ^3^Dept of Cardiology, Haga Hospital, The Hague, The Netherlands; ^4^Dept of Clinical Pharmacy and Pharmacology, University Medical Center Groningen, Groningen, The Netherlands; ^5^Dept of Physiology, Institute of Cardiovascular Research, VU University Medical Center, Amsterdam, The Netherlands; ^6^Dept of Cardiology, University Medical Center St. Radboud, Nijmegen, The Netherlands; ^7^Dept of Cardiology, Amphia Hospital, Breda, The Netherlands; ^8^Dept of Pediatrics, Division of Pediatric Cardiology, Erasmus Medical Center – Sophia Children’s Hospital, Rotterdam, The Netherlands; ^9^Dept of Cardiology, Bravis Hospital, Rosendaal, The Netherlands; ^10^Dept of Cardiology, Medisch Spectrum Twente, Enschede, The Netherlands; ^11^Dept of Cardiology, VU University Medical Center, Amsterdam, The Netherlands; ^12^Dept of Cardiology, Catharina Hospital, Eindhoven, The Netherlands;

